# Evolutionary Games of Low-Carbon Behaviors of Construction Stakeholders under Carbon Taxes

**DOI:** 10.3390/ijerph18020508

**Published:** 2021-01-09

**Authors:** Qiang Du, Yunqing Yan, Youdan Huang, Chanchan Hao, Jiao Wu

**Affiliations:** 1 School of Economics and Management, Chang’an University, Xi’an 710064, China; q.du@chd.edu.cn (Q.D.); huangyoudan@chd.edu.cn (Y.H.); cchan_hao@163.com (C.H.); jiao_wu@chd.edu.cn (J.W.); 2 Center for Green Engineering and Sustainable Development, Xi’an 710064, China

**Keywords:** low-carbon buildings, carbon tax, evolutionary game, low-carbon behaviors

## Abstract

The development of low-carbon buildings (LCBs) in China has not reached its expected status, although the Chinese government has formulated many relevant regulations. The real estate developers and consumers are essential participants in the development of LCBs. This paper explores whether the government’s implementation of the carbon tax will change their choices of LCBs. Evolutionary game models between developers and consumers are established under static and dynamic carbon taxes. Their evolutionarily stable strategies (ESS) are deduced in different situations. According to the real scenarios in China, numerical simulations are further conducted to show that carbon tax influences the low-carbon behaviors of stakeholders in the construction industry. Under a static carbon tax, the two players cannot reach an equilibrium state, while the game system is stable under a dynamic tax. The probability of the developers constructing LCBs is positively related to the carbon tax, while its degree is gradually weakened as the tax rate increases. Therefore, an appropriate tax should be set to promote the development of LCBs effectively. Finally, policy implications are put forwarded to guide the participants’ low-carbon behaviors and reduce the carbon emissions in the Chinese construction industry.

## 1. Introduction

The construction industry exerts essential economic, environmental and social impacts in China. With the rapid urbanization process, the industry has become one of the main contributions to carbon emissions due to the large consumption of resources and materials. It has caused adverse effects on the environment [1]. Low carbon buildings (LCBs) gradually turn into a development tendency, which is considered to reduce carbon emissions, improve energy efficiency, and use low-carbon materials, technologies and renewable energies. Since the Chinese government promulgated the corresponding guidelines for the development of low-carbon and energy-saving buildings, green buildings accounted for more than 40% of newly built civil buildings in cities and towns according to statistics. The development of LCBs needs to be improved to achieve the expected status [2].

The major participants in LCB development are construction enterprises, consumers and the government [2,3]. Specifically, construction enterprises tend to be risk averse and profit driven. They often measure costs and benefits in the short term and do not pay attention to energy conservation and emission reduction due to higher carbon costs [4]. For consumers, the market price of buildings is still a significant factor, although operating costs and living comfort are also taken into consideration with the increase in low-carbon awareness [5]. The government is the primary driver of LCB development, stimulating and regulating industry participants to take relevant actions for LCBs. They together constitute the joint driving force for the low-carbon transformation of the construction industry [6].

The different interests or needs of stakeholders result in various behaviors. Exploring the low carbon behaviors of them is significant to facilitate LCB development. In academics, researchers focus on providing basic research and data by field investigations and questionnaire surveys to analyze the current status of existing stakeholders [7]. For instance, Zhang et al. [2] explored the factors of imperfect development of LCBs in Chinese cities using a questionnaire survey. Further, they explored the impact of critical factors on enterprises’ low-carbon willingness by structural equation models. However, it is also vital to describe and analyze the decision-making behaviors of the subjects in different situations more clearly from the theoretical level and numerical simulation.

The evolutionary game theory provides a useful research framework for social behavior and its influences on environmental issues [8,9]. The low carbon and sustainable development of the construction industry is a dynamic and complicated process. With the passage of time and changes in revenue, each participant is bounded rational in the system. Their behaviors are dynamically evolving, and they continue to learn, compete and adapt. Existing literature gradually apply this dynamic and systematic method to the exploration of incentive mechanisms for the development of green buildings [10,11]. Studies with LCBs as the research object, considering the impact of carbon tax policy on the low carbon behavior of construction enterprises and consumers, have done little.

This paper constructs the evolutionary game model of the developers and homebuyers under static and dynamic carbon tax policy. Major players’ behavioral strategies and interactions in different situations and incentive mechanisms are systematically analyzed. It is conducive to obtain a better understanding of the underlying mechanism of the low-carbon transformation in the construction industry, to guide the low-carbon behavior of enterprises and consumers, and to provide a sound rationale for police makers proposing a carbon tax.

The remainder of this paper is arranged as follows. Section 2 presents the literature reviews. In Section 3, evolutionary game models under static and dynamic carbon tax policies are formulated and analyzed. In Section 4, the related theoretical analyses are verified by a simulation of an illustrative example. The results are discussed in Section 5. Conclusions and suggestions are provided in Section 6.

## 2. Literature Review

The literature related to this study consists of three parts: low-carbon behaviors in the construction industry, the effects of carbon tax policy, and the application of evolutionary games.

### 2.1. Low Carbon Behaviors in the Construction Industry

With the imperative of energy conservation and emission reduction, relevant researches have been conducted on the low-carbon behaviors of construction practitioners. From the perspective of builders, Osmani et al. [12] discussed the feasibility of zero-carbon homes and noted that housebuilders face numerous barriers to delivering zero carbon homes, such as financial, technical, cultural and legislative barriers. The green procurement practices adopted by developers are significant for the promotion of green building material products. Based on this, Shen et al. [13] investigated the green procurement behavior of developers in a real estate development set. Onuoha et al. [14] used structural equation modeling methods to identify the motivating factors that affect the decisions of developers and investors to invest in green commercial real estate.

Concerning consumers, the willingness to buy green buildings is a research hotspot. For example, Zalejska-Jonsson [15] explored occupants’ willingness to pay for green apartments in Sweden and showed that a 5% premium for low-energy buildings is a reasonable decision. Portnov et al. [16] indicated an acceptable price premium of the green building between 7% and 10% through a national online survey in Israel. In addition to quantifying the residents’ willingness to pay, some scholars have also proposed that improving the performance of green residential buildings and providing more reliable and concrete public information would increase the willingness of buyers to purchase green housing [17]. Martek et al. [18] also focused on end-users, who are important to sustainability projects and provided the research on the roles of the interests and impacts of end-users. However, few researchers have explored the low-carbon behavior choices of developers and homebuyers, that is, the interactive effect when choosing traditional buildings (CBs) or LCBs. In this study, we study the factors that affect their behaviors by analyzing their profits and strategic evolution, thus encouraging them to choose LCBs.

### 2.2. The Effects of Carbon Tax Policy

The government plays a vital role in promoting the construction industry’s sustainable development using legal and incentive economic systems. Li et al. [19] used the environmental-economic simulation model to assess the carbon emissions reduction potential of China’s iron and steel industry, and they introduced both environmental policies and technological upgrades to optimize. Cadavid-Giraldo et al. [20] evaluated the effectiveness of the carbon tax mechanism in encouraging sustainable cement production. They studied the impact of different carbon emission prices on the decision to reduce emissions in the cement supply chain. Shi et al. [21] discussed the possible impact of different carbon tax conditions on the energy consumption and macroeconomics of China’s construction industry. The results showed that a reasonable carbon tax rate varies with the construction stage.

In recent years, most studies tend to explore the impact of carbon tax policy on the environment and economy from the national level and the provincial level by establishing a calculable general equilibrium (CGE) model [22]. Khastar et al. [23] used the CGE model to establish links between carbon taxes and economic indicators, production and consumption sectors and assessed the impact of carbon taxes on the Finnish industry structure. Due to the varied types and amounts of energy consumption by different industries, some researchers have proposed a differential carbon tax. They focus on finding a balance point between the sustainable development of the industry and the realization of carbon emission reduction targets [21,24].

In addition to the macro impact on the industry, carbon prices can further enable companies to adjust business strategies by adjusting carbon costs to restrain emissions effectively. Yenipazarli [25] examined the effect of carbon taxes on optimal production and pricing decisions and explored the impact of remanufacturing and social-related factors on the firm-level profit balance. Kuo et al. [26] studied the effects of the carbon tax on enterprises’ investments in new technology. They indicated that appropriate tax levels would prompt enterprises to change their production processes. Wang et al. [27] found that a carbon tax can facilitate the innovation and sharing of low-carbon technologies among enterprises to some extent.

The above studies use different perspectives and theoretical methods to investigate the influence of carbon tax policy on industry and enterprises, which have provided references and guidance for our work. The carbon tax has been viewed as an essential policy instrument for mitigating carbon emissions and stimulating the low carbon decision-making behavior of enterprises [28,29]. Therefore, this research introduces the carbon tax policy in the construction industry and studies the impact of carbon tax policies on the behavior of micro-subjects. It aims to stimulate developers and homebuyers to select LCBs, thereby promoting the development of LCBs.

### 2.3. The Application of the Evolutionary Game

The evolutionary game theory combines Lamarck’s genetic theory with Darwin’s biological evolution theory, and is an available tool for analyzing interactions between different participants. When faced with complex situations, the player’s rationality is limited, and the best strategy cannot be determined at the beginning of the game. However, players can imitate others or learn to adjust and optimize their strategies [8]. Therefore, its bounded rationality hypothesis seems to be more realistic than traditional game theory. This method effectively explains the evolutionary paths and reasons according to which groups reach equilibrium by analyzing the dynamic evolutionary process.

Evolutionary game theory is widely used in the impact of emission reduction policies on the low-carbon behavior of enterprises. For example, Fan et al. [30] constructed evolutionary game models based on agents of the government and enterprises in the cases of a lack of supervision and supervision. The optimal strategy for supervising low-carbon subsidies is studied. Mahmoudi [31] established an evolutionary game model between government and producers based on three objective functions, aiming to analyze government policies’ impact on producer behavior and carbon emissions.

In the construction industry, existing researches used evolutionary games to study the quantitative effects of incentive policies for the promotion of green buildings, the green retrofits of buildings, etc. [10,11]. The government and developers or contractors are usually selected as game players. Recently, some scholars have also considered the consumer factor. Cohen et al. [32] used game theory to explain the obstacles to energy conservation and emission reduction in Israel’s construction industry. They predicted that subsidies could encourage builders and buyers to improve energy efficiency. Lu et al. [6] established a multiparty evolutionary game model, considering government reputation costs and consumer low-carbon compensation factors. The research has laid the foundation for exploring the inherent laws of the low-carbon transition of construction companies theoretically and methodically.

However, considering the guiding significance of carbon policies for production and emission reduction, it is still unclear how carbon tax policy work and to what extent it can affect the stakeholders in the construction industry. Based on the abundant previous studies, this paper utilizes the evolutionary game to identify the decision-making behaviors of developers and homebuyers under carbon tax policy in LCBs management. It will not only help the government to formulate practical carbon emission policies to promote the construction market from the traditional model to the low-carbon model but also lead enterprises and consumers in low-carbon behavior choices.

In addition, most researchers have assumed that the parameter settings of policies are constant and static. Only a few regard policies as variables [8,33,34]. Similarly, this paper explores the interaction mechanism between game players under two scenarios, static and dynamic carbon tax, which will be favorable for comprehensive and systematic analysis. Different from those studies previously mentioned, our study’s contributions lie in the following: (1) an examination of the effect of the carbon tax on the stakeholders’ behaviors in the construction industry, (2) the comparison of static and dynamic tax in the evolutionary game process, and (3) a reference for the government to establish appropriate carbon policy, aiming at promoting the low-carbon development of the construction market.

## 3. The Evolutionary Game Model

The evolutionary game models between developers and homebuyers under different incentive scenarios are established in this section. First, we analyze the evolutionarily stable strategy (ESS) of developers and homebuyers under a static carbon tax model with different constraints. Next, the mixed strategies chosen by the players under a dynamic carbon tax policy are discussed.

### 3.1. Model Assumptions

#### 3.1.1. Players and Strategies

The game is composed of two players: developers and homebuyers. The developers implement different project positioning decisions, constructing low carbon buildings (LCBs) or conventional buildings (CBs), while the homebuyers choose to buy LCBs or CBs. Therefore, the different strategy combinations of developers and homebuyers are as follows: (Construct LCB, Buy LCB), (Construct LCB, Buy CB), (Construct CB, Buy LCB), and (Construct CB, Buy CB). Moreover, we propose the following assumptions: both players are limited rational economic persons, and they each have only two different strategies, each of which is mutually exclusive. Even if the low-carbon strategies of the players are inconsistent, they can reach a deal. Based on the market situation, the developers have certain dominant advantage, which will be described in detail in the following profit parameter explanation.

#### 3.1.2. Payoffs

Cost and price: *C*_1_ and *C*_0_ represent the cost of developers when they develop LCBs and CBs, respectively, assuming $C_{1}>C_{0}$ and $\Delta C=C_{1}-C_{0}$. According to the previous literature, developers face incremental costs when developing LCBs because they need to invest some capital for researching and developing low-carbon technologies. The prices of LCBs and CBs are *P*_1_ and *P*_0_, respectively $P_{1}>P_{0}$. The building price is the benefit gained by the developer and the cost incurred by the homebuyer. 

If a homebuyer wants to buy a CB, but there is none in the market, he or she will accept an LCB at a lower price. He or she will pay only *P*_0_. When homebuyers take interest in LCBs, but developers construct only CBs, the homebuyers will have to purchase ordinary houses. Simultaneously, assuming that the consumer will receive pollution compensation *B* from developers [6]. This compensation is regarded as the developer transferring part of the proceeds from non-low-carbon construction to consumers, which will ensure that the consumers’ willingness to consume LCBs are not affected.

Environmental benefits: *β* represents homebuyers’ ecological awareness

(1) β∈[0,1]

*E* denotes environmental value, the positive impact of LCBs on the environment compared with CBs [32]. When homebuyers initially buy CBs, they may ignore the environment, so the *β* value is 0. Once developers construct the CBs, their energy-conservation and emissions-reduction are negligible; thus, the variable *E* is 0.

Subsidies: The government’s incentive and constraint mechanisms play a significant role in the construction industry and affect developers’ and consumers’ strategies in the process of developing LCBs. Developers adopting low-carbon technologies to construct LCBs can gain governmental subsidy *S*_1_, and homebuyers who buy LCBs can achieve governmental subsidy *S*_2_.

Carbon tax: The carbon tax is based on carbon emissions and tax rate, which are calculated directly on the basis of fossil fuel consumption and emission coefficients [35]. It is assumed that the government imposes a carbon tax on only developers that generate more carbon emissions than acceptable, expressed as follows:

(2) $$T_{i}=\{\begin{array}{cc} (e_{i}-e_{0})\times Ct & e_{i}\geq e_{0}\\ 0 & e_{i}<e_{0} \end{array}i=CB,LCB$$

In Equation (2), *e*_0_ represents carbon emissions at the acceptable level, and *e_LCB_* and *e_CB_* denote carbon emissions produced during the construction phase of LCBs and CBs, respectively. The carbon emissions of CBs are higher than *e*_0_, while those of LCBs are lower. *T_i_* represents the carbon tax, and *Ct* denotes the tax rate. According to the above assumption, only a carbon tax on developers who build CBs is considered; thus,

(3) $$T=T_{CB}=(e_{CB}-e_{0})\times Ct\;\;\;T\geq 0$$

The carbon tax for developers who construct CBs will become their internalized cost so that developers may transfer part of the carbon tax to homebuyers. Assume that the proportion of the assigned tax is recorded as *α* (*α* ∈ [0, 1]), which means that homebuyers will face the increased cost, *αT*, when they buy CBs. As mentioned above, homebuyers will gain compensation from the developers in situations in which their desire to buy LCBs is frustrated. We assume that the compensation is relative to the shifted carbon tax, and it is written as B=αT. This assumption simplifies the model and distinguishes it from the strategy in which the players choose conventional buildings. To some extent, the set potentially encourages the low-carbon behavior of consumers.

In this study, the parameters related to price are considered per square meter. The definition of all parameters is uniform and listed in Table 1.

### 3.2. Static Model Description and Analysis

#### 3.2.1. Basic Model

At the primary stage, supposing that the proportion of developers choosing to construct LCBs is *x* (x∈[0,1]), the ratio of developers who choose to build CBs is 1 − *x*. Similarly, *y* (y∈[0,1]) is the ratio of homebuyers buying LCBs, and 1 − *y* is the proportion of homebuyers purchasing CBs. During the game process, players will adjust their strategies when their payoffs are under the average; thus, *x* and *y* are changing. According to the above relationships between two players and the basic assumptions, the payoff matrix is depicted in Table 2.

(4) $$\left\{\pi_{D1},\;\pi_{H1}\right\}=\left\{P_{1}-C_{1}+S_{1},\;-P_{1}+S_{2}+\beta \cdot E\right\}$$

(5) $$\left\{\pi_{D2},\;\pi_{H2}\right\}=\left\{P_{0}-C_{1}+S_{1},\;-P_{0}\right\}$$

(6) $$\left\{\pi_{D3},\;\pi_{H3}\right\}=\left\{P_{0}-C_{0}-T,\;-P_{0}\right\}$$

(7) $$\left\{\pi_{D4},\;\pi_{H4}\right\}=\left\{P_{0}-C_{0}-T+\alpha T,\;-P_{0}-\alpha \cdot T\right\}$$

Let *U_D_*_1_ and *U_D_*_2_ represent the expected profits of “Construct LCB” and “Construct CB” for developers, respectively. Based on Table 2, these profits can be expressed as follows:

(8) $$U_{D1}=y\pi_{D1}+(1-y)\pi_{D2}$$

(9) $$U_{D2}=y\pi_{D3}+(1-y)\pi_{D4}$$

The average profit of developers is denoted as $\bar{U_{D}}$ which is obtained as follows:

(10) $$\bar{U_{D}}=xU_{D1}+(1-x)U_{D2}$$

Let *U_H_*_1_ and *U_H_*_2_ represent the expected profits of “Buy LCB” and “Buy CB” for homebuyers, respectively. Based on Table 2, these profits can be expressed as follows:

(11) $$U_{H1}=x\pi_{H1}+(1-x)\pi_{H3}$$

(12) $$U_{H2}=x\pi_{H2}+(1-x)\pi_{H4}$$

The average profit of homebuyers is written as $\bar{U_{H}}$ which is obtained as follows:

(13) $$\bar{U_{H}}=yU_{H1}+(1-y)U_{H2}$$

According to Friedman et al. [36] and Xiao et al. [37], the replicator dynamic equations of the constructing LCBs strategy selected by developers (*F*(*x*)) and the buying LCBs strategy adopted by homebuyers (*F*(*y*)) respectively, are obtained as follows:

(14) $$F(x)=dx/dt=x(U_{D1}-\bar{U_{D}})=x(1-\;x)[(1-\alpha )T-\Delta C+S_{1}+y(\Delta P+\alpha T)]$$

(15) $$F(y)=dy/dt=y(U_{H1}-\bar{U_{H}})=y(1\;-\;y)[\alpha T+x(-\Delta P+S_{2}+\beta E-\alpha T)]$$

#### 3.2.2. Model Analysis

The adjustment speeds of developers and homebuyers’ behavioral strategies are expressed by Equations (14) and (15), respectively. The game will reach a relatively stable equilibrium state when these replication factor dynamic equations are zero. Let dx/dt=0 and dy/dt=0; we obtain four fixed equilibrium points: (0,0), (1,1), (0,1) and (1,0). When $\pi_{H1}<\pi_{H2}$,$\pi_{D2}<\pi_{D4}$ and $\pi_{D1}<\pi_{D3}$, that is, $-\Delta P+S_{2}+\beta E<0$ and $\Delta P+\alpha T>\Delta C-S_{1}-(1-\alpha )T>0$, we obtain the fifth equilibrium point (*x*_0_, *y*_0_):

(16) $$x_{0}=\frac{-\alpha T}{-\alpha T-\Delta P+S_{2}+\beta E},\;y_{0}=\frac{\Delta C-S_{1}-(1-\alpha )T}{\Delta P+\alpha T}$$

The stability of equilibrium points can be analyzed by the Jacobian matrix. The type of equilibrium points can be judged by calculating the determinant *det*(*J*) and trace *tr*(*J*) of the matrix [36]. When the equilibrium point of the combination of Equations (13) and (14) satisfies the condition $det(J)=|\begin{array}{cc} a & b\\ c & d \end{array}|=ad-bc>0$ and tr(J)=a+d<0, the equilibrium point is an ESS; when det(J)>0 and tr(J)>0, it is an unstable point; when det(J)<0, and tr(J)=0 or uncertainty, it is a saddle point. The Jacobian matrix *J* composed of the above replicator dynamic equations is as follows:

(17) $$J=\left[\begin{array}{cc} \left(1-2x)[(1-\alpha )T-\Delta C+S_{1}+y(\Delta P+\alpha T)\right] & x(1-x)(\Delta P+\alpha T)\\ y(1-y)(-\Delta P+S_{2}+\beta E-\alpha T) & \left(1-2y)[\alpha T+x(-\Delta P+S_{2}+\beta E-\alpha T)\right] \end{array}\right]$$

Substituting the above five equilibrium points into the Jacobian matrix, Table 3 shows the calculations of their *det*(*J*) and *tr*(*J*).

Based on Equations (4)–(7), $\pi_{H3}>\pi_{H4}$; thus, the kinds of equilibrium points depend on the symbols $\pi_{H1}-\pi_{H2}$, $\pi_{D2}-\pi_{D}{}_{4}$ and $\pi_{D1}-\pi_{D3}$. There are six situations, and Table 4 analyses the local stability of the equilibrium points of the game in different situations.

**Figure 1:**
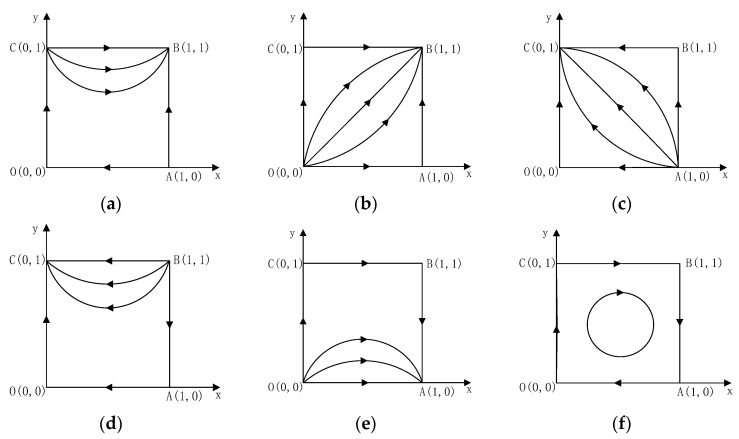
Dynamic evolutionary diagrams: (**a**) Situation 1; (**b**) Situation 2; (**c**) Situation 3; (**d**) Situation 4; (**e**) Situation 5; (**f**) Situation 6.

Situation 1: $S_{2}+\beta E>\Delta P$, and $\Delta P+\alpha T>\Delta C-S_{1}-(1-\alpha )T>0$. Figure 1a displays the evolutionary process of developers and homebuyers’ behavior strategies. Table 4 and Figure 1a show that (1,1) is the ESS of the game; (Construct LCB, Buy LCB) is the ESS of the game between developers and homebuyers.

Situation 2: $S_{2}+\beta E>\Delta P$, and $\Delta C-S_{1}-(1-\alpha )T<0$. Table 4 and Figure 1b also show that (1,1) is the ESS of the game in condition 2, which means that (Construct LCB, Buy LCB) is the ESS.

Situation 3: $S_{2}+\beta E>\Delta P$, and $\Delta C-S_{1}-(1-\alpha )T>\Delta P+\alpha T$. Regarding the game in situation 3, Table 4 and Figure 1c show that the point (0,1) is the ESS, namely (Construct CB, Buy LCB) is the ESS.

Situation 4: $S_{2}+\beta E<\Delta P$, and $\Delta C-S_{1}-(1-\alpha )T>\Delta P+\alpha T$. Table 4 and Figure 1d also show that (0,1) is the ESS of the game in situation 4, which means that (Construct CB, Buy LCB) is the ESS.

Situation 5: $S_{2}+\beta E<\Delta P$, and $\Delta C-S_{1}-(1-\alpha )T<0$. Table 4 and Figure 1e show the dynamic evolutionary process. Under this situation, the ESS of the game is (1,0), namely (Construct LCB, Buy CB).

Situation 6: $S_{2}+\beta E<\Delta P$, and $\Delta P+\alpha T>\Delta C-S_{1}-(1-\alpha )T>0$. Table 4 and Figure 1f display the two-dimensional coordinate graphs of the relationship between the two game parties and the evolutionary path. Under situation 6, there is no ESS, and the evolutionary path appears as a closed-loop.

Table 4 and Figure 1 show that under the situation of $S_{2}+\beta E>\Delta P$, and $\Delta P+\alpha T>\Delta C-S_{1}-(1-\alpha )T$, all the ESS are (Construct LCB, Buy LCB). It is an ideal state. Increasing the subsidies, the carbon tax, the external environmental benefits of LCBs and consumers’ awareness of the LCB concepts, and decreasing the cost of low-carbon technologies will have a positive impact on the low-carbon behavioral choices of developers and consumers. The players will actively make the decisions to choose LCBs when they obtain sufficiently high additional benefits from LCBs. In situations 3 and 4, for both $S_{2}+\beta E<\Delta P$ and $S_{2}+\beta E>\Delta P$, provided that $\Delta C-S_{1}-(1-\alpha )T>\Delta P+\alpha T$ is satisfied, (Construct CB, Buy LCB) is the ESS. This result means that regardless of whether consumers are interested in LCBs, developers always choose to build ordinary houses. However, based on the above assumption, once developers construct CBs, homebuyers have to buy them. For this situation, it can be understood that LCBs have not yet been accepted and fully recognized by most developers in the real world.

Under situation 5, (Construct LCB, Buy CB) is the only ESS. In contrast to the previous situations, homebuyers tend to buy CBs, but they accept LCBs. This result indicates that consumers’ low-carbon awareness is not strong. In reality, developers and consumers are likely to encounter cost-benefit mismatches when choosing LCBs; specifically, the dwellers enjoy all benefits during the use phase while developers pay all the upfront costs [38]. In addition, the transaction game between developers and homebuyers is complicated. The players’ strategies are affected by various factors, such as information asymmetry, the severe inequality in the bargaining power of developers and home buyers during the market transaction [39], and many external uncertainty factors.

There is no ESS in the last situation; the four saddle points and a central point are obtained. The central point (*x*_0_, *y*_0_) satisfies the elementary condition of the ESS, so it is the Lyapunov stability. The Lyapunov stability has two stable attributes: indifferent and asymptotic stabilities. If it is asymptotically stabled, point (*x*_0_, *y*_0_) is the ESS. We further discuss its stability. Consider the point (*x*_0_, *y*_0_), the Jacobian matrix *J*′ is valued as:

(18) $$J^{\prime }=\left[\begin{array}{cc} 0 & \frac{-\alpha T(-\Delta P+S_{2}+\beta E)(\Delta P+\alpha T)}{{\left(-\Delta P+S_{2}+\beta E-\alpha T\right)}^{2}}\\ \frac{\left[\Delta C-S_{1}-(1-\alpha )T\right]\left[\Delta P+\alpha T-\Delta C+S_{1}-(1-\alpha )T\right](-\Delta P+S_{2}+\beta E-\alpha T)}{{\left(\Delta P+\alpha T\right)}^{2}} & 0 \end{array}\right]$$

The Eigenvalues of Jacobian matrix *J*′ can be deduced as:

(19) $$\lambda_{1,2}=\pm \frac{i\sqrt{\left[\Delta C-S_{1}-(1-\alpha )T\right]\left[\Delta P+\alpha T-\Delta C+S_{1}-(1-\alpha )T\right](-\Delta P+S_{2}+\beta E-\alpha T)\left[-\alpha T(-\Delta P+S_{2}+\beta E)(\Delta P+\alpha T)\right]}}{\left(\Delta P+\alpha T)(-\Delta P+S_{2}+\beta E-\alpha T\right)}$$

where *λ*_1_ and *λ*_2_ are virtual characteristic roots, so the point (*x*_0_, *y*_0_) is not asymptotically stabilities. Therefore, in this situation, this system does not have an ESS, and any subtle changes may have a significant impact on the behaviors of developers and homebuyers. The government should put forward relevant measures to change this situation. The last situation is discussed in depth below.

### 3.3. Dynamic Model Description and Analysis

According to the above analysis, there is no ESS between developers and homebuyers under the static carbon tax in the situation $S_{2}+\beta E<\Delta P$ and $\Delta P+\alpha T>\Delta C-S_{1}-(1-\alpha )T>0$. When the original value is changed, the evolutionary game will obtain the corresponding ESS. We study the mixed-strategy chosen by developers and homebuyers under the dynamic policy in this section.

This study introduces a dynamic carbon tax, and the taxation is changed from the initial constant *T* to T(x)=(1−x)T, where *T* denotes the maximum carbon tax [8]. The carbon tax is related to the probability *x* that developers construct LCBs. As the probability (1 − *x*) that developers will construct CBs is higher, the government will gradually increase the carbon tax.

Substituting (1−x)T for *T* into Equations (14) and (15), the dynamic replication equation of the “Construct LCB” strategy chosen by developers is as follows:

(20) $$F(x)=dx/dt=x(1-\;x)[(1-\alpha )T(x)-\Delta C+S_{1}+y(\Delta P+\alpha T(x))]$$

Similarly, the dynamic replication equation of the “Buy LCB” strategy chosen by homebuyers is as follows:

(21) $$F(y)=dy/dt=y(1\;-\;y)[\alpha T(x)+x(-\Delta P+S_{2}+\beta E-\alpha T(x))]$$

Letting the dynamic replication equations be dx/dt=0 and dy/dt=0, we obtain the equilibrium points (0, 0), (0, 1), (1, 0) and (1, 1); when $x_{1}\in [0,1]$, $y_{1}\in [0,1]$, we can obtain (*x*_1_, *y*_1_):

(22) $$x_{1}=\frac{\Delta P-S_{2}-\beta E+2\alpha T-\sqrt{\left(\Delta P-S_{2}-\beta E)(\Delta P-S_{2}-\beta E+4\alpha T\right)}}{2\alpha T}$$

(23) $$y_{1}=\frac{2\alpha T(\Delta C-S_{1})-(1-\alpha )T\left[-\Delta P+S_{2}+\beta E+\sqrt{\left(\Delta P-S_{2}-\beta E)(\Delta P-S_{2}-\beta E+4\alpha T\right)}\right]}{\alpha T\left[\Delta P+S_{2}+\beta E+\sqrt{\left(\Delta P-S_{2}-\beta E)(\Delta P-S_{2}-\beta E+4\alpha T\right)}\right]}$$

The Jacobian matrix *J*_1_ of the replicator dynamic equations is:

(24) $$J_{1}=\left[\begin{array}{cc} \left(1-2x)[S_{1}-\Delta C+(1-\alpha )T(x)+y(\Delta P+\alpha T(x)]+x(1-x)[(1-a)T^{\prime }(x)+\alpha T^{\prime }(x)y\right] & x(1-x)[\Delta P+\alpha T(x)]\\ y(1-y)[-\Delta P+S_{2}+\beta E-\alpha T(x)+(1-x)\alpha T^{\prime }(x)] & \left(1-2y)[(1-x)\alpha T(x)+x(-\Delta P+S_{2}+\beta E)\right] \end{array}\right]$$

The stability analysis of *J*_1_ under $\Delta P+\alpha T>\Delta C-S_{1}-(1-\alpha )T>0$, $S_{2}+\beta E<\Delta P$, is shown in Table 5. According to the stability analysis of the game under a dynamic carbon tax situation, points (0, 0) and (0, 1) are saddle points, points (1, 0) and (1, 1) are uncertain points, and (*x*_1_, *y*_1_) is an asymptotic stable point.

For point (*x*_1_, *y*_1_), the Jacobian matrix *J*_1_′ is valued as:

(25) $${J_{1}}^{\prime }=\left[\begin{array}{cc} -\frac{\varphi (2\alpha T-\varphi )\left[\Delta P(1-\alpha )T+\alpha T(\Delta C-S_{1})\right]}{2{\left(\alpha T\right)}^{2}{\left(2\Delta P+\varphi \right)}^{2}} & \frac{\varphi (2\alpha T-\varphi )(2\Delta P+\varphi )}{8{\left(\alpha T\right)}^{2}}\\ \frac{\left[2\alpha T(\Delta C-S_{1})-(1-\alpha )T\varphi \right]\left[2\alpha T\Delta P+\alpha T\varphi -2\alpha T(\Delta C-S_{1})+(1-\alpha )T\varphi \right](-\sqrt{\left(\Delta P-S_{2}-\beta E)(\Delta P-S_{2}-\beta E+4\alpha T\right)})}{{\left(\alpha T\right)}^{2}{\left(2\Delta P+\varphi \right)}^{2}} & 0 \end{array}\right]$$

where

(26) $$\varphi =-\Delta P+S_{2}+\beta E+\sqrt{\left(\Delta P-S_{2}-\beta E)(\Delta P-S_{2}-\beta E+4\alpha T\right)}$$

The eigenvalues of Jacobian matrix *J*_1_′ can be deduced as:

(27) $$\lambda_{3,4}=\frac{\varphi (\varphi -2\alpha T)\left[\Delta P(1-\alpha )T+\alpha T(\Delta C-S_{1})\right]\pm i\sqrt{\Omega }}{4{\left(\alpha T\right)}^{2}{\left(2\Delta P+\varphi \right)}^{2}}$$

where *λ*_3_ and *λ*_4_ are complex numbers with a negative real component and Ω > 0. Thus, the point (*x*_1_, *y*_1_) is asymptotic stability. 

In conclusion, there is an ESS in the evolutionary game between developers and homebuyers under the dynamic tax. It implies that the probability of developers constructing LCBs is *x*_1_, and the ratio of homebuyers buying LCBs is *y*_1_. As mentioned in the previous section, if the political power on the games works, the initial value of this system changes and the evolutionary path will be from (*x*_1_, *y*_1_) to (1, 1).

## 4. Numerical Simulation Analysis

### 4.1. Parameter Values

A numerical example based on an actual case study is provided to demonstrate the theoretical results. The relevant data in the model are given considering the real situation of the Chinese construction industry and based on the collected information and relevant reviews. 

Since the existing qualitative research on the LCBs market is still limited, we have to mention green buildings. It is generally believed that green buildings contain many practices and technologies, reducing the negative impact on resource consumption, the environment and human health, and carbon emissions are also lower than ordinary buildings [16,40]. Therefore, we introduced some parameters related to green buildings. The initial value settings and their source description are presented in Table 6. Other parameters comprehensively consider the construction company’s production cost data and social welfare, ∆*P* = 2.6 × 10^2^ CNY/m2, *S*_2_ = 0.4 × 10^2^ CNY/m^2^, *α* = 0.5. To simplify the model simulation, some parameters are limited to one decimal and the unit is united as “10^2^ CNY/m^2^”. For example, 100 CNY/m^2^ = 1 × 10^2^ CNY/m^2^.

In addition, the settings of the two initial probabilities of the participants need to be explained, *x* = 0.5 and *y* = 0.5. According to the 2014–2020 National Plan on New Urbanization states that China’s urban green buildings will account for 50% of new buildings by 2020. It is assumed that the probability that construction companies will choose the “Construct LCB” strategy in the initial state is 0.5. Similarly, although some researchers have studied the willingness to pay for green buildings, and different regions, genders, ages, education levels may affect their desire. Because the proportion of consumers who are willing to buy low-carbon buildings is not clear, the initial state of public consumers choosing LCBs is assumed to be 0.5. The evolutionary game models are simulated and analyzed using MATLAB (The MathWorks, Inc., Natick, MA, USA)

### 4.2. Simulation Results

#### 4.2.1. The Strategies of Players under Static Carbon Tax

Based on the above analyzes of the static model, the two players have different evolutionarily stable strategies under different income relations. Among them, (Construct LCB, Buy LCB) is an ideal stable strategy combination. Take “Situation 1” as an example. The behavioral evolution paths of both parties are simulated. 

**Figure 2:**
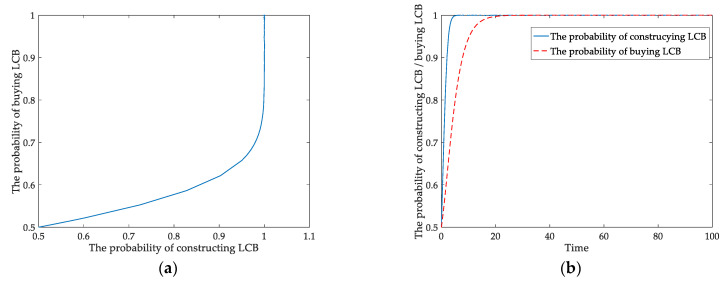
Evolutionary process under static model: (**a**) behavioral strategies; (**b**) the probabilities of behavior.

When the income relationship satisfies $S_{2}+\beta E>\Delta P$, $\Delta P+\alpha T>\Delta C-S_{1}-(1-\alpha )T>0$, the results are shown in Figure 2a,b. As the iteration process proceeds, the probabilities of developers constructing LCBs and homebuyers buying LCBs increase. When the sum of subsidy and environmental benefit is higher than the incremental price of LCB, the interests of homebuyers are guaranteed, and they are willing to buy LCBs. When the net income of LCBs (ΔP−ΔC), government subsidy and tax are positive, the developers have enough revenue incentives to build LCBs. Finally, their ESS is (Construct LCB, Buy LCB). The system evolves from (0.5, 0.5) towards the stable equilibrium point (1, 1).

Satisfying the above simulation conditions, this study continues to simulate the influence of important parameters on the evolutionary results, e.g., incremental price of LCBs, government subsidies, the low-carbon technology cost and home buyers’ low-carbon awareness.

**Figure 3:**
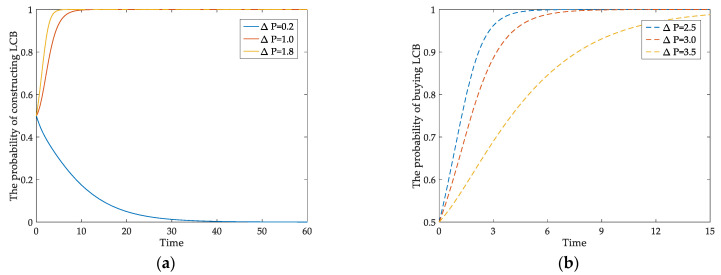
The impact of Δ*P* on (**a**) developer’ behavioral evolution; (**b**) homebuyers’ behavioral evolution.

The impacts of the incremental price of LCBs compared to CBs (Δ*P*) on developers’ and homebuyers’ behaviors are shown in Figure 3a,b separately. In the case of initial conditions *x* = 0.5, and with the parameter Δ*P* changes from 0.2 to 1.8, the developers will eventually choose to build LCBs and the probability changes from 0 to 1. When the value of Δ*P* increases from 2.5 to 3.5, the probability of consumers buying LCBs tends to be 1, while the speed is lower. The incremental price of LCBs will play a significant role in their behaviors. Higher incremental income is benefit for developers but not good for consumers. There has always been a game about housing prices between them. Excessive low-carbon house prices will discourage homebuyers’ low-carbon willingness, and the market prospects of developers will be uncertain. Therefore, an appropriate price mechanism can effectively encourage them to choose LCBs.

**Figure 4:**
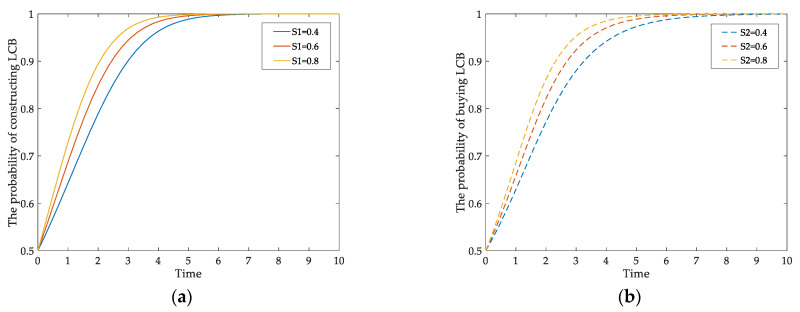
The impact of *S*_1_ and *S*_2_ on: (**a**) developer’ behavioral evolution; (**b**) homebuyers’ behavioral evolution.

Figure 4a,b respectively show the influence of government subsidy incentives on the behaviors of developers and homebuyers. When the parameters *S*_1_ and *S*_2_ change from 0.4 to 0.8, the behavioral probabilities of both players tend to 1 from the initial 0.5. From the simulation results, it can be seen that the time when the probabilities of the two players reach 1 is different. The probability of the developers is one at *t* = 6, and the consumers’ is at *t* = 8. The comparison shows that the government’s incentives to market entities can speed up their choices of LCBs and the effects of subsidies on them are different.

**Figure 5:**
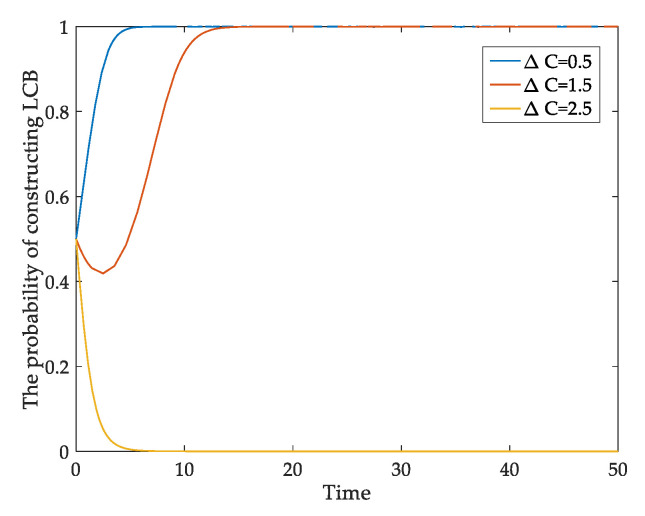
The impact of Δ*C* on developers’ behavioral evolution.

**Figure 6:**
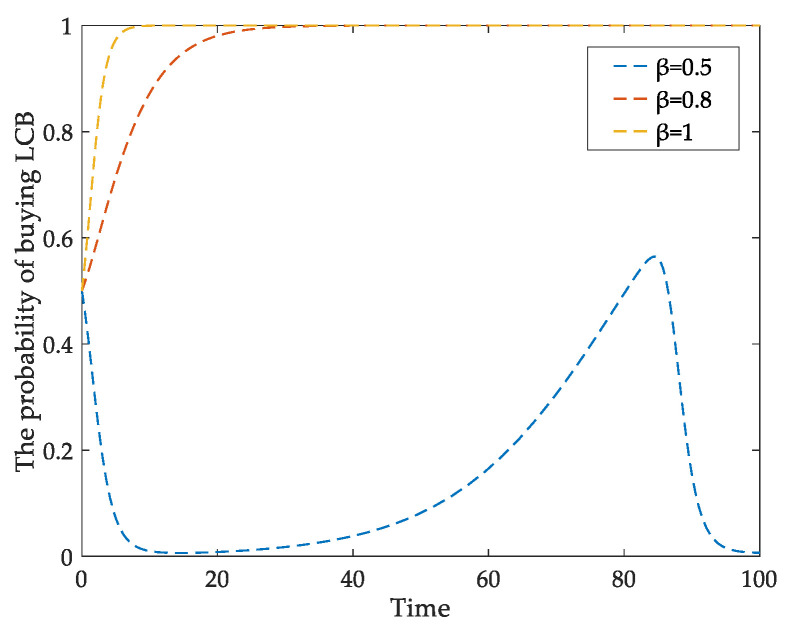
The impact of *β* on homebuyers’ behavioral evolution.

For developers, one of the most important factors hindering their low-carbon transition is the additional technical cost of LCBs. Therefore, Figure 5 simulates the impact of Δ*C* on the low-carbon behavior of developers. When Δ*C* changes from 0.5 to 2.5, the developer’s behavioral probability of constructing LCBs changes from 1 to 0, it is essential to reduce developers’ high cost constraints to promote low-carbon construction. In addition, the public’s recognition and acceptance of LCBs are also important. Figure 6 shows the effect of homebuyers’ low-carbon awareness on their LCBs purchase behavior. When *β* = 0.5, the probability of buying LCBs is unstable. When *β* = 0.8, homebuyers are willing to buy LCBs over time. With the increase of *β* value, the probability of buying LCBs approaches one faster. By promoting the LCBs’ advantages continuously, the consumers’ low-carbon awareness can be improved, which will effectively stimulate them to purchase LCBs.

#### 4.2.2. The Mixed Strategies of Players under Different Scenarios

According to the previous theoretical derivation, for situation 6, which is more realistic, there are two different system evolution trends in different scenarios. In this section, the interactions between developers and buyers in the evolutionary game are displayed intuitively under static and dynamic carbon tax policies. They all seek optimal strategies by always imitating and learning both parties’ strategies.

**Figure 7:**
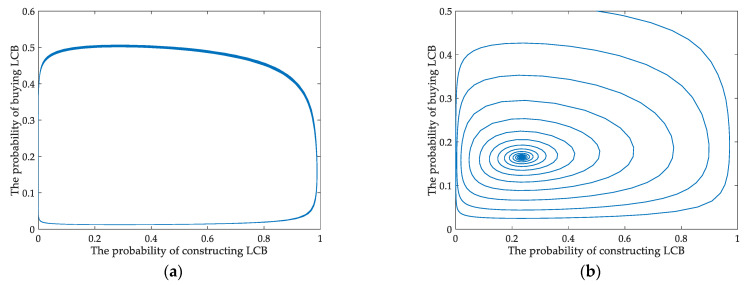
Evolutionary path of strategies (**a**) under static carbon tax (**b**) under dynamic carbon tax.

In Figure 7a, the behavioral strategy’s evolutionary path between developers and homebuyers presents a closed-loop under the static policy. The results indicate that there is no Nash equilibrium between them. During the starting stage of the construction industry’s low-carbon transformation, the Chinese government tried to provide static tax to cultivate the LCB market. However, it could not make appropriate tax policy adjustments based on developers’ behaviors in static situations. Figure 7b shows that the curves’ course approaches a focal point of gradual stability from the initial point of (0.5, 0.5) throughout the cycle. The simulation results confirm the theoretical research. The findings verify that (*x*_1_, *y*_1_) is the center of asymptotic stability. Thus, the evolutionary game will reach equilibrium when the government levies a dynamic carbon tax on developers.

#### 4.2.3. The Behaviors of Players under Different Scenarios

**Figure 8:**
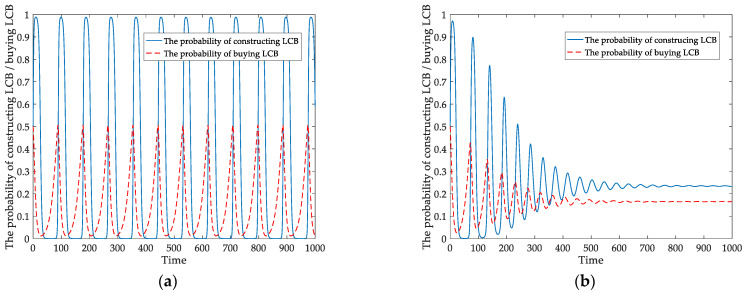
Players’ behaviors in an evolutionary game (**a**) with a static carbon tax (**b**) with a dynamic carbon tax.

Under different carbon tax policies, we intuitively examine the strategies chosen by developers and homebuyers. Figure 8a,b present how static and dynamic carbon tax affect the behavioral probabilities of developers and homebuyers.

Figure 8a shows the probabilities of developers constructing LCBs and homebuyers buying LCBs under the static carbon tax. The solid line denotes the probability of developers constructing LCBs; the dotted line represents the probability of homebuyers buying LCBs. The probabilities of them are continually fluctuating. It also means that the game system is not stable.

The probabilities of developers constructing LCBs and homebuyers buying LCBs under the dynamic carbon tax mechanism are displayed in Figure 8b. The probability fluctuation amplitude of developers constructing LCBs gradually decreases. The solid line is approximately stable at time *t* = 800 and *x* = 0.23. Similarly, the probability of homebuyers buying LCBs will reach a steady state after a short time fluctuation, *y* = 0.16, and the dotted curve comes stability at time *t* = 600. This result means that the evolutionary game system gradually becomes stable and that LCB enterprises’ groups reach a certain scale. Comparing Figure 8a with Figure 8b, we discover that the fluctuation amplitude of the two players’ behavior probability gradually decreases under the dynamic carbon tax. The dynamic policy is effective compared with the static system in stimulating developers to construct LCBs.

#### 4.2.4. The Evolutionary Path of the Behavior of Developers under Different Carbon Tax Rates (Ct)

**Figure 9:**
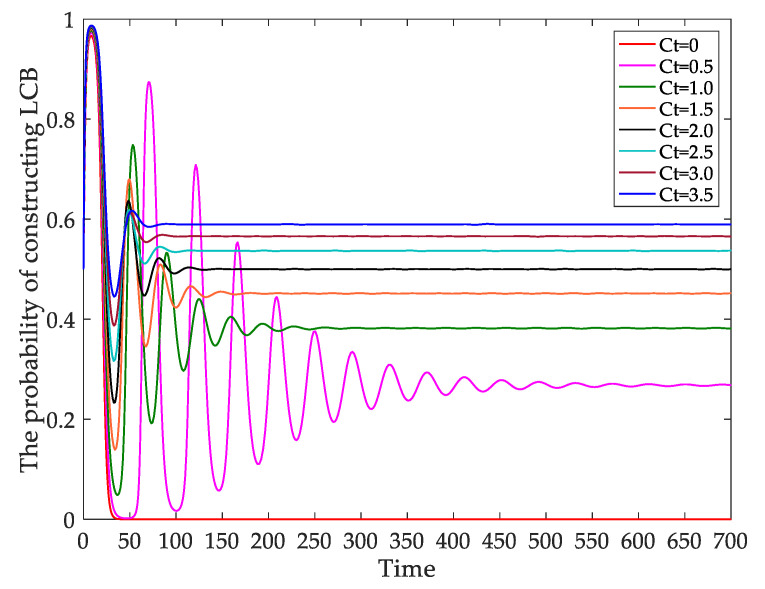
Evolutionary path of the behavior strategies of developers under different dynamic carbon tax rates.

In this section, the influence of varying carbon tax rates (dynamic policy) on the behaviors of developers’ decision-making is examined. The influence of different carbon tax rates on the behaviors of developers constructing LCBs is presented in Figure 9. When *Ct* = 0, the probability of developers constructing LCBs is zero. As the value of *Ct* increases, the probability of developers constructing LCBs is increasing. When *Ct* = 0.5, 1.0, 1.5, 2.0, 2.5, 3.0, and 3.5, the probability of developers constructing LCBs is approximately stable at time *t* = 520, 240, 150, 120, 100, 80, 80. When *Ct* = 3.0 and *Ct* = 3.5, the time that the probability of developers constructing LCBs being stable is almost the same. It can also be seen that when *Ct* = 2.0, the probability is the same as the initial set value, namely *x* = 0.5. When the value of *Ct* exceeds 2.0, the increment of probability is no longer obvious.

In the beginning stage, the behaviors of developers fluctuate, and the fluctuation in amplitude gradually decreases over time. Eventually, the evolutionary paths of developers tend to be stable. With the increase in *Ct*, the restriction on developers constructing CBs is working. The probability of developers choosing LCBs increases, and the time to stability is faster. However, when the carbon tax rate is raised to a certain level, the increasingly smaller time difference over the probability tending to be stable can be determined, and the increase in the probability becomes lower.

## 5. Discussion

The low-carbon transformation of the construction industry involves multiple stakeholders, such as governments, construction enterprises and homebuyers. In the low-carbon development of the construction industry, developers often respond to a series of signals such as government policies and consumer demand. Therefore, this study provides insights into how they can make decisions under different policies and discusses the carbon tax’s effectiveness on the construction industry’s low-carbon development.

Under the static carbon tax, multiple ESSs can be obtained if the initial value has been changed, as shown in Figure 1. The evolutionary behaviors between developers and homebuyers vary with different constraints. Figure 1a and Figure 2, when the conditions $\Delta P+\alpha T>\Delta C-S_{1}-(1-\alpha )T$ and $S_{2}+\beta E>\Delta P$ are met, the ideal state (Construct LCB, Buy LCB) is the ESS. Only when all stakeholders find that incremental investments in “going green” are financially viable will they voluntarily adopt green practices. Whether a construction company adopts a low-carbon development strategy depends on the measurement of its benefits and costs. To reduce the cost of low-carbon technology investment and obtain more benefits, developers should focus on the application of renewable energy technological innovations and increase the utilization of low-carbon materials and technologies. In addition, they actively cooperate with the government to guide consumers to buy LCBs, thereby expanding the market. For consumers, it is imperative to improve consumers’ low-carbon awareness by promoting the superiority of LCBs. Providing subsidies or home loan discounts can enhance the incentives for purchasing LCBs, which will directly stimulate a virtuous circle of low-carbon production for construction enterprises,

In the actual situation, the static carbon tax cannot make the game system between developers and consumers stable, as shown in Figure 1f, Figure 7a and Figure 8a. We introduce a dynamic carbon tax for further analysis using the tax policy as a decision variable related to the probability of the players’ behavior to reflect the evolutionary process better. As analyzed and simulated above, the evolutionary game has an asymptotic stability point under the dynamic policy, determined from Figure 7b. It may be due to the government implementing a static carbon tax in the initial stage. Nevertheless, it cannot adjust the policy in a dynamic and timely way based on developers’ behaviors under the static carbon tax. The developers will be short of elasticity and rely on financial incentives. It is visually found that the behavioral probabilities of the players continuously fluctuate under the static carbon tax by comparing Figure 8a,b. In contrast, their behavioral probability curves tend to be stable from fluctuations under dynamic tax. Therefore, from the perspective of the evolutionary path of the players, we consider a dynamic carbon tax more effective than a static one in promoting developers and homebuyers to choose LCBs.

Figure 9 shows that the probability of developers constructing LCBs is zero without a carbon tax and is positively correlated with the carbon tax. The results indicate that the carbon tax will affect the low carbon behaviors of developers. Because the government uses the carbon tax to intervene in micro-subjects for emission reduction by changing prices so that the external costs of enterprises with high carbon emissions are internalized. When the profits from developing CBs are lower than those from developing LCBs, more developers will construct LCBs. Therefore, levying a carbon tax is feasible in the construction industry and promotes developers’ choice of LCBs.

Based on the simulation results of related parameters, it is relatively reasonable to set *Ct* at 200 CNY/ton. When the tax rate is less than 200 CNY/ton, the probability of developers constructing LCBs is less than 0.5. When the tax rate continuously increases from 200 to 350 CNY/ton, the behavior probability of developers for constructing LCBs only increases by less than 0.1. Low tax rates have no obvious incentive for developers, especially when developers compare lower carbon costs with higher profits. As the tax rate continuously increases, the degree of increase in the probability of constructing LCBs is weakened. Once the taxation reaches a certain threshold, it will start to inhibit the further diffusion of LCB instead of stimulating it. Although the high tax rate will impose a considerable cost burden on developers, consumers will be likely to become the ultimate undertakers. So, the role of taxation will be limited at that time. Moreover, LCBs development’s goal cannot be achieved only by raising the carbon tax rate and the carbon price. The carbon tax is dependent on the carbon price and carbon emissions. Only by adopting the related measures to energy conservation and emission reduction can LCBs be effectively promoted.

## 6. Conclusions

To facilitate the development of LCBs and to mitigate construction carbon emissions, the evolutionary game models between developers and homebuyers under two scenarios, static and dynamic carbon taxes, are established. It focuses on the evolutionary path of the game between two players and the impact of the carbon tax on their behaviors. An illustrative example based on the Chinese construction industry is examined by simulation. Based on the theoretical analysis and simulation results, we draw the following conclusions.

(1) Under static policy conditions, multiple ESSs can be obtained when the initial values change. The ideal state (building LCB, purchasing LCB) will be an ESS when developers and consumers get higher profits from LCBs than CBs. The process of choosing LCBs will be accelerated by increasing government incentives, reducing the incremental cost of LCBs, controlling excessive real estate market prices and improve the homebuyers’ low-carbon awareness.

(2) Taking into account the reality, the evolutionary processes of the game between developers and homebuyers are different under static and dynamic carbon taxes. There is no evolutionary stability strategy under static conditions, while it is stable under dynamic conditions. The probability of the developers constructing LCBs is positively related to the carbon tax, but the degree is gradually weakened as the tax rate increases. Therefore, a reasonable carbon tax rate will better promote the low-carbon development of the construction industry.

### Policy Implications

Macro-level management is one of the significant factors affecting emission reduction in constructions. The promulgation of a series of codes such as “Code for Quality Inspection of Building Energy Conservation Projects” and “Standard for energy efficiency inspection in residential buildings” has played a vital role in reducing emissions. The government’s incentive schemes can promote the development of LCBs, such as increasing certification of LCBs and providing subsidies for low-carbon research and development of enterprises. To further optimize and control emissions and energy consumptions, the government should promote the application of low-carbon technologies in life cycle construction process, e.g., BIM digital technology, modular prefabrication, and so on.

Besides, the government can choose the right opportunity to levy a carbon tax for the construction industry. Many scholars have explored the feasibility of taxation to achieve carbon emission reduction. Some countries have adopted a carbon tax as an effective emission reduction policy for sustainable development. Combining this research, some suggestions for the central government to formulate an extra carbon tax policy are put forward in terms of the scope, the link and the tax rate of collection.

First, the introduction of carbon tax needs to be coordinated with carbon trading policy to avoid double pressure on companies implementing two policies simultaneously. The carbon trading market currently involves mostly energy-intensive industrial enterprises. Carbon taxes can be levied on construction enterprises and others outside the carbon trading system.

Second, the collection of carbon tax can be divided into production and consumption. The initial taxation to construction enterprises could reduce the cost and difficulty of collecting and monitoring. However, the emissions generated by consumers cannot be ignored due to long-term residence and use. Energy saving and emission reduction can be facilitated by influencing consumer behavior in the later period.

Third, to encourage developers to construct LCBs, reasonable carbon tax rates need to be set. The option of the tax rate needs to consider factors such as the benefits of the participating entities, emission status and so on. With the development of the low-carbon construction market, the carbon tax rate should be adjusted dynamically.

In addition, it is vital for local governments to seriously respond to policy implementation and regulate the behavior of enterprises, which will prevent consumers from becoming the ultimate recipients and avoid the weakening of the original significance of the policy. The emission monitoring in the construction industry should also be carried out to check the emission reduction effects of enterprises.

In this study, the choice behaviors of developers and homebuyers for LCBs under different carbon tax policies are simulated and discussed. Simultaneously, the impact of the carbon tax on low-carbon behaviors is demonstrated. Nevertheless, there are some limitations to this study. For instance, because the carbon tax has not been implemented in China and specific studies related to the benefits of LCBs are limited, most of the simulation data derive from regulation documents and previous literature. Bringing the experimental data into the simulation model and conducting a correlation analysis remains to be performed. Also, in reality, it is difficult for the government to adjust the carbon tax at any time, and ways to implement the dynamic carbon tax should be further explored.

## Figures and Tables

**Table 1:** Parameters setting.

Parameters	Descriptions
*C*_1_	The cost of developers constructing LCB
*C*_0_	The cost of developers constructing CB
∆*C*	Incremental cost ($\Delta C=C_{1}-C_{0}$); The cost of implementing the low-carbon technologies
*P*_1_	The price of LCB
*P*_0_	The price of CB (without regard to a carbon tax)
∆*P*	Incremental price ($\Delta P=P_{1}-P_{0}$)
*β*	Consumers’ environmental awareness
*E*	Friendly environmental and ecological value
*B*	Developers’ pollution compensation for homebuyers
*S*_1_	Subsidy for developers who construct LCB
*S*_2_	Subsidy for homebuyers who buy LCB
*e*_0_	Carbon emissions at the acceptable level
*e_LCB_*	Carbon emissions produced by building LCB
*e_CB_*	Carbon emissions produced by building CB
*Ct*	Carbon tax; the price of carbon emission per ton
*T*	Carbon tax on developers who construct CB
*α*	The proportion of the carbon tax taken by homebuyers

**Table 2:** Payoff matrix between the developers and homebuyers.

Developers	Homebuyers	
	Buy LCB (*y*)	Buy CB (1−*y*)
Construct LCB (*x*)	$\pi_{D1}$,$\pi_{H1}$	$\pi_{D2}$,$\pi_{H2}$
Construct CB (1−*x*)	$\pi_{D3}$,$\pi_{H3}$	$\pi_{D4}$,$\pi_{H4}$

**Table 3:** The *det*(*J*) and *tr*(*J*) of five equilibrium points.

Equilibrium Point	*det*(*J*)	*tr*(*J*)
(0, 0)	$(\pi_{D2}-\pi_{D4})\left(\pi_{H3}-\pi_{H4}\right)$	$(\pi_{D2}-\pi_{D4})+\left(\pi_{H3}-\pi_{H4}\right)$
(0, 1)	$-(\pi_{D1}-\pi_{D3})\left(\pi_{H3}-\pi_{H4}\right)$	$(\pi_{D1}-\pi_{D3})-\left(\pi_{H3}-\pi_{H4}\right)$
(1, 0)	$-(\pi_{D2}-\pi_{D4})\left(\pi_{H1}-\pi_{H2}\right)$	$-(\pi_{D2}-\pi_{D4})+\left(\pi_{H1}-\pi_{H2}\right)$
(1, 1)	$(\pi_{D1}-\pi_{D3})\left(\pi_{H1}-\pi_{H2}\right)$	$-(\pi_{D1}-\pi_{D3})-\left(\pi_{H1}-\pi_{H2}\right)$
(*x*_0_, *y*_0_)	$-\frac{(\pi_{H4}-\pi_{H3})\left(\pi_{H1}-\pi_{H2}\right)(\pi_{D4}-\pi_{D2})\left(\pi_{D1}-\pi_{D3}\right)}{\left(\pi_{H1}-\pi_{H2}-\pi_{H3}+\pi_{H4})(\pi_{D1}-\pi_{D2}-\pi_{D3}+\pi_{D4}\right)}$	0

**Table 4:** Local stability analysis of equilibrium points.

**Equilibrium Point**	**Situation 1**			**Situation 2**			**Situation 3**		
	***det***	***tr***	**State**	***det***	***tr***	**State**	***det***	***tr***	**State**
(0, 0)	−	N	Saddle point	+	+	Unstable point	−	N	Saddle point
(0, 1)	−	N	Saddle point	−	N	Saddle point	+	−	ESS
(1, 0)	+	+	Unstable point	−	N	Saddle point	+	+	Unstable point
(1, 1)	+	−	ESS	+	−	ESS	−	N	Saddle point
(*x*_0_, *y*_0_)	meaningless								
**Equilibrium Point**	**Situation 4**			**Situation 5**			**Situation 6**		
	***det***	***tr***	**State**	***det***	***tr***	**State**	***det***	***tr***	**State**
(0, 0)	−	N	Saddle point	+	+	Unstable point	−	N	Saddle point
(0, 1)	+	−	ESS	−	N	Saddle point	−	N	Saddle point
(1, 0)	−	N	Saddle point	+	−	ESS	−	N	Saddle point
(1, 1)	+	+	Unstable point	−	N	Saddle point	−	N	Saddle point
(*x*_0_, *y*_0_)	meaningless						+	0	central point

“+” signifies greater than 0, “−” signifies less than 0, “N” signifies uncertainty.

**Table 5:** Stability analysis of the evolutionary game under the dynamic carbon tax.

Equilibrium Point	Symbol of *det*(*J*)	Symbol of *tr*(*J*)	Results
(0, 0)	-	N	Saddle point
(0, 1)	-	N	Saddle point
(1, 0)	N	N	Uncertain point
(1, 1)	N	N	Uncertain point
(*x*_1_, *y*_1_)	N	-	Asymptotic stable point

**Table 6:** Initial values of the parameters and description.

Parameter	Description
∆*C* = 1.3 × 10^2^ CNY/m^2^	According to the data in the 2015 National Green Building Evaluation Mark Statistical Report, the incremental cost of three-star green buildings averages 135.92 CNY/m^2^.
*S*_1_ = 0.8 × 10^2^ CNY/m^2^	In 2012, the central government introduced an incentive scheme for the Chinese Green Building Label, which stipulated that the government would give a subsidy of 80 CNY /m^2^ to developers constructing three-star green buildings.
*e_CB_* = 0.5 ton	An ordinary building releases approximately 0.5 ton CO_2_e/m^2^ throughout its life cycle [41].
*e*_0_ = 0.1 ton	Based on the *Stern Review of Climate Change Economics*, to avoid exceeding the natural ability of the Earth to remove greenhouse gas, emissions must be reduced by 80% from the current level. Hence, LCBs should reduce emissions by at least 80% compared to CBs. Therefore, acceptable carbon emissions are $e_{0}=0.5\times (1-80\%)=0.1$.
*Ct* = 0.4 × 10^2^ CNY/ton	According to the *Framework Design of China’s Carbon Taxation*, the carbon tax would be imposed starting in 2012, and the carbon tax rate may reach 40 CNY/ton in 2020.
*β* = 1	From the initial assumption and parameter setting, it can be observed that homebuyers’ environmental awareness *β* appears in the strategy of (Construct LCB, Buy LCB). The low-carbon behaviors of consumers are closely related to their low-carbon awareness, so the value of *β* is set to 1 when homebuyers buy LCBs.

## Data Availability

No new data were created or analyzed in this study. Data sharing is not applicable to this article.
